# Keynote 3: Professor Adrian BaumanSystems approaches to physical activity – becoming commonplace or still a rare occurrence?

**DOI:** 10.1093/eurpub/ckae114.003

**Published:** 2024-09-26

## Abstract

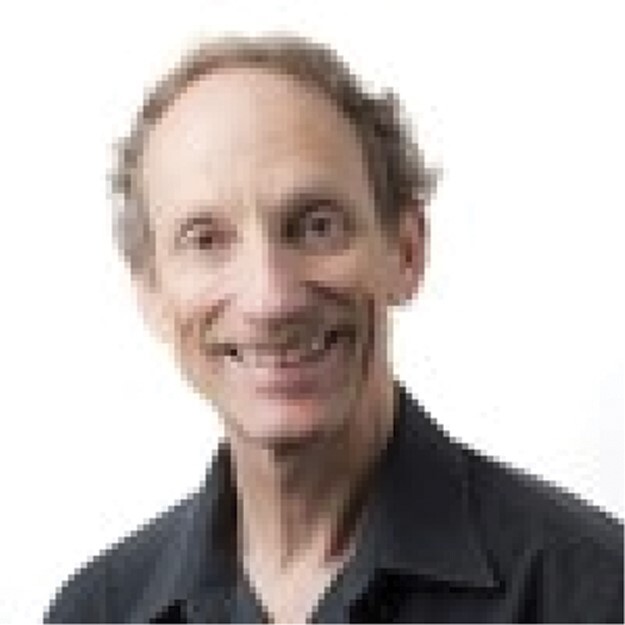

Systems thinking is becoming a popular concept in planning population approaches to physical activity. These approaches are not new but build upon earlier thinking epitomised in the Healthy Public Policy component of the 1986 Ottawa Charter for Health Promotion and in the subsequent WHO 'Health in all Policies' frameworks. Most recently systems underpinning and describe the 2018 WHO Global Action Plan on Physical Activity, with work proposed in the intersecting areas of: active societies, active environments, active people and active systems.

This approach acknowledges that physical activity occurs within social, environmental and policy sets of interacting systems. The system is composed of multiple attributes, requires extensive partnerships across agencies and sectors, and incorporates complex program delivery and evaluation methods. Initial stages in systems approaches include the development of system-wide maps of physical activity in a community or region. These maps help agencies to develop a shared understanding of the influences on physical activity and identify opportunities and priorities for action. However, the systems map is only the beginning of the process, and are not useful unless followed by systems-level planning, resourcing, and implementing action. Other methods to understand systems include *system dynamic models*, which demonstrate predicted physical activity outcomes, given different types and combinations of interventions. System work often occurs at the municipality or city-level, and exploratory research can inform policymakers of barriers or facilitators encountered; this may facilitate the development of solutions. Finally, systems approaches take years to implement, but eventually should be assessed for their impact on physical activity prevalence.

For governments, agencies or regions with a defined physical activity plan and a long-term commitment to implementing physical activity programs, systems thinking offers an integrated framework and the potential to influence physical activity levels through actions in both health and non-health sectors.

